# Advanced gastric cancer: CT radiomics prediction and early detection of downstaging with neoadjuvant chemotherapy

**DOI:** 10.1007/s00330-021-07962-2

**Published:** 2021-04-28

**Authors:** Qinmei Xu, Zeyu Sun, Xiuli Li, Chen Ye, Changsheng Zhou, Longjiang Zhang, Guangming Lu

**Affiliations:** 1 Department of Medical Imaging, Jinling Hospital, Nanjing University School of Medicine, Nanjing, 210002 Jiangsu China; 2 Deepwise AI Lab, Deepwise Inc., No. 8 Haidian avenue, Sinosteel International Plaza, Beijing, 100080 China; 3 Research Institute of General Surgery, Jinling Hospital, Nanjing, 210002 Jiangsu China

**Keywords:** Machine learning, Neoadjuvant chemotherapy, Gastric cancer, Decision-making

## Abstract

**Objectives:**

To develop and evaluate machine learning models using baseline and restaging computed tomography (CT) for predicting and early detecting pathological downstaging (pDS) with neoadjuvant chemotherapy in advanced gastric cancer (AGC).

**Methods:**

We collected 292 AGC patients who received neoadjuvant chemotherapy. They were classified into (a) primary cohort (206 patients with 3–4 cycles chemotherapy) for model development and internal validation, (b) testing cohort I (46 patients with 3–4 cycles chemotherapy) for evaluating models’ predictive ability before and after the complete course, and (c) testing cohort II (*n* = 40) for model evaluation on its performance at early treatment. We extracted 1,231 radiomics features from venous phase CT at baseline and restaging. We selected radiomics models based on 28 cross-combination models and measured the areas under the curve (AUC). Our prediction radiomics (PR) model is designed to predict pDS outcomes using baseline CT. Detection radiomics (DR) model is applied to restaging CT for early pDS detection.

**Results:**

PR model achieved promising outcomes in two testing cohorts (AUC 0.750, *p* = .009 and AUC 0.889, *p* = .000). DR model also showed a good predictive ability (AUC 0.922, *p* = .000 and AUC 0.850, *p* = .000), outperforming the commonly used RECIST method (NRI 39.5% and NRI 35.4%). Furthermore, the improved DR model with averaging outcome scores of PR and DR models showed boosted results in two testing cohorts (AUC 0.961, *p* = .000 and AUC 0.921, *p* = .000).

**Conclusions:**

CT-based radiomics models perform well on prediction and early detection tasks of pDS and can potentially assist surgical decision-making in AGC patients.

**Key Points:**

*• Baseline contrast-enhanced computed tomography (CECT)-based radiomics features were predictive of pathological downstaging, allowing accurate identification of non-responders before therapy.*

*• Restaging CECT-based radiomics features were predictive to achieve pDS after and even at an early stage of neoadjuvant chemotherapy.*

*• Combination of baseline and restaging CECT-based radiomics features was promising for early detection and preoperative evaluation of pathological downstaging of AGC.*

**Supplementary Information:**

The online version contains supplementary material available at 10.1007/s00330-021-07962-2.

## Introduction

Advanced gastric cancer (AGC) stands for 50–80% of all cases of gastric cancer (GC) [1]. The major amount of tumors (35–51%) fail to achieve pathological downstaging (pDS) after neoadjuvant chemotherapy and tumor progression was commonly observed (15%) [2, 3]. Therefore, early and accurate patient stratification would be helpful to select good candidates for neoadjuvant treatment of AGC patients.

Computed tomography (CT) is routinely used for tumor monitoring over the course of treatment [4]. Baseline contrast-enhanced CT (CECT) is the preferred imaging examination to diagnose the TNM stage for gastric cancer before neoadjuvant chemotherapy in clinical practice. Restaging CECT evaluates tumor downstaging after neoadjuvant chemotherapy [4]. However, patients at the same stage on baseline CT can display diverse chemosensitivities. Low sensitivity (37–50%) of tumor size-based measurement [5] and inaccuracy (37–57%) of tumor restaging [6] by visual assessment are reported.

Radiomics [7] defines quantitative imaging feature extraction that facilitates exploration of radiological heterogeneity. CT-based radiomics analysis was useful in stage prediction and therapeutic selection for AGC patients [8–10], as well as in predicting response to chemotherapy [9, 10]. For example, pre-treatment CT texture analysis can provide information regarding the response rate to neoadjuvant therapy for GC [9]. Radiomics analysis shows differences between responders and non-responders to chemotherapy [10]. However, for AGC patients with neoadjuvant chemotherapy, accurate prediction of pDS is yet to be elucidated. Furthermore, the value of CT-based radiomics for early detection of achieving pDS, a critical biomarker for timely treatment decision-making, has not been explicitly addressed.

In this study, we seek to develop CT radiomics models for prediction and early detection of pDS to neoadjuvant chemotherapy in AGC. Specifically, our first goal is to examine the radiomics value using baseline CECT for predicting pDS before neoadjuvant chemotherapy. Our second goal is to assess restaging CECT for early detection of pDS after the start of chemotherapy.

## Materials and methods

### Patient enrollment and population

**Fig. 1:**
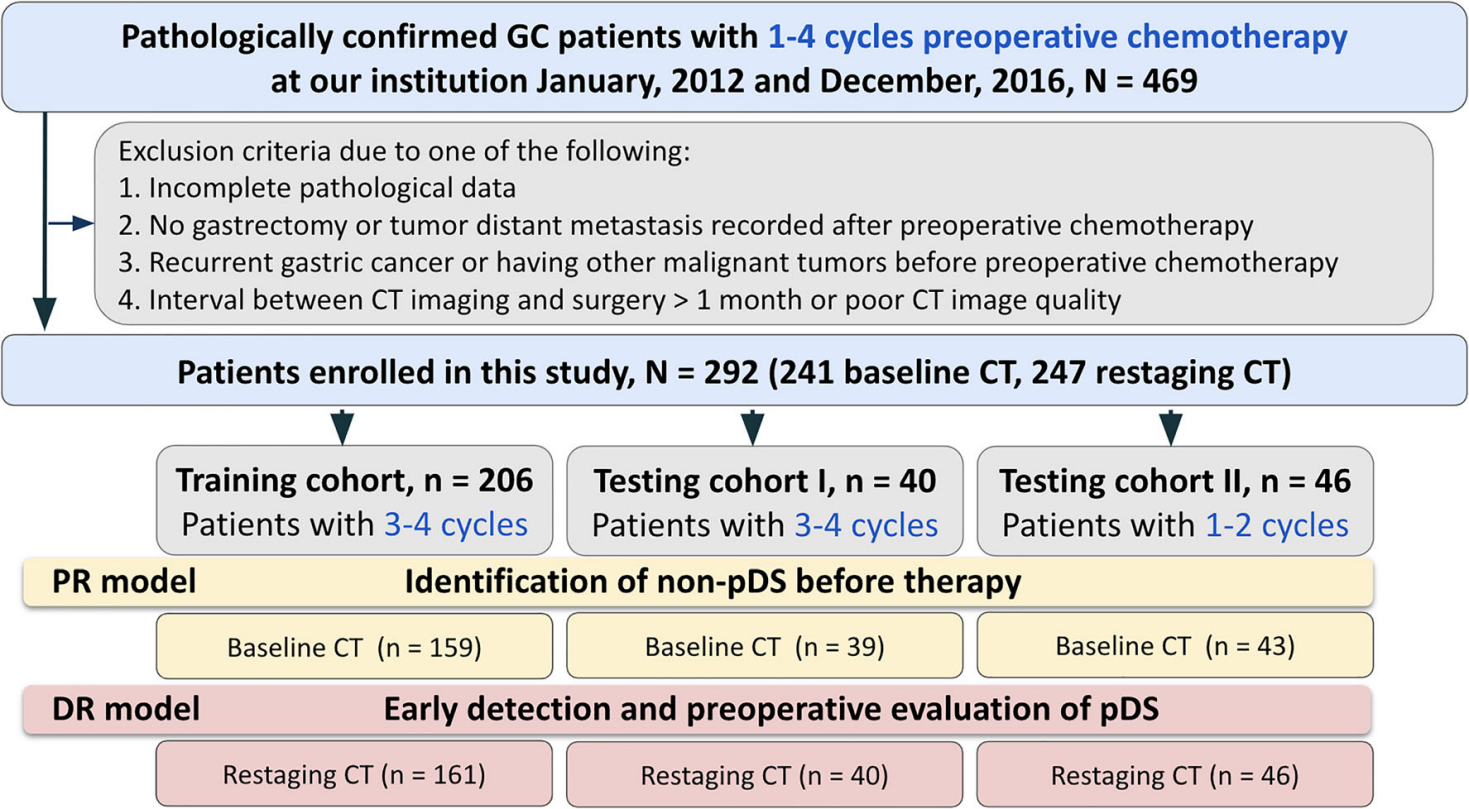
Workflow of the proposed study. Note: N, number; GC, gastric cancer; CT, computed tomography; pDS, pathological downstaging; PR, prediction radiomics model; DR, detection radiomics model

We collected 469 histologically confirmed GC patients (clinical stage cT3/4N0/+M0 on admission) who received chemotherapy and followed by surgery in Jinling Hospital between Jan. 2012 and Dec. 2016 (Fig. 1). The inclusion criterion was patients with histologically confirmed gastric cancer and absence of distant metastases on admission, who underwent 1–4 cycles of neoadjuvant chemotherapy followed by gastrectomy with lymph node dissection at our institution or had distant metastasis after chemotherapy. The exclusion criteria were as follows: (1) an interval of more than 1 month between CT imaging and surgery; (2) neither gastrectomy nor tumor distant metastasis was recorded after neoadjuvant chemotherapy; (3) recurrent gastric cancer or having other malignant tumors before neoadjuvant chemotherapy; (4) incomplete pathological data; (5) poor CT image quality. Then two experienced radiologists (Q.M.X. and C.S.Z., with 4 and 9 years of experience in gastric CT imaging, respectively) evaluated the image artifacts and the degree of the gastric filling (Figure S1) on each baseline or restaging CT image to ensure image quality for image segmentation and feature extraction (Fig. 1, the last step in the exclusion criteria). Table S1 displays the image quality evaluation criterion and the number of patients in each grade. Finally, 292 patients with 241 baseline CT (primary cohort, *n* = 159; testing cohort I, *n* = 39; testing cohort II, *n* = 43) and 247 restaging CT (primary cohort, *n* = 161; testing cohort I, *n* = 40; testing cohort II, *n* = 46) were used for radiomics analysis. The institutional review board approved this retrospective investigation and was in line with the Health Insurance Portability and Accountability Act. The need for informed patient consent was waived.

### Study design

**Fig. 2:**
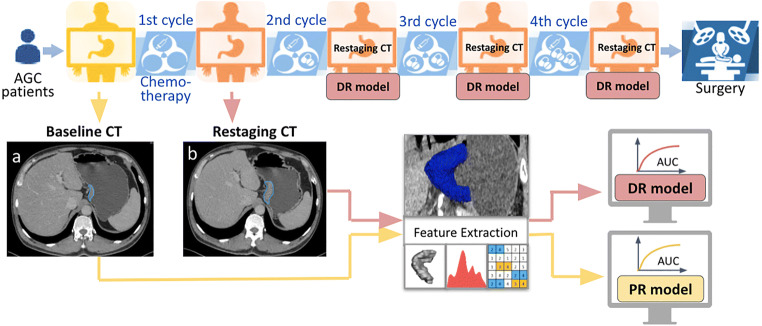
Illustration of the proposed study in the position of GC neoadjuvant chemotherapy timeline. (**a**) and (**b**) are portal venous phase baseline and restaging CT images of a female patient, at 47 years old with AGC. The segmented tumor contours were outlined by blue curves. Note: AGC, advanced gastric cancer; CT, computed tomography; pDS, pathological downstaging; PR, prediction radiomics model; DR, detection radiomics model

We split the cohort into three subsets: one primary cohort and two testing cohorts (Fig. 1). Patients in the primary cohort (*n* = 206, Jan. 2012–Dec. 2015) and testing cohort I (*n* = 40, from Jan. 2016–Dec. 2016) received a complete course of neoadjuvant chemotherapy (with 3–4 cycles) and followed by surgery. Patients in testing cohort II (*n* = 46, from Jan. 2012–Dec. 2016) underwent early trial of dissection due to cessation of chemotherapy (with 1–2 cycles). We used the primary cohort for radiomics models’ development and internal validation, while testing cohort I was for evaluating models’ predictive ability before and after the complete course, and testing cohort II was especially for assessing model performance at an early stage of the treatment course. The prediction radiomics (PR) model built upon the baseline CT (taken on admission) aimed at prediction of pDS before therapy (Fig. 2). The detection radiomics (DR) model based on the restaging CT (done post-chemotherapy) was for early detection of pDS after the start of neoadjuvant chemotherapy (Fig. 2). We also compared the ability of the DR model with clinical routine RECIST (Response Evaluation Criteria in Solid Tumors) method [11] in two testing cohorts.

### CT image acquisition parameters

Patients took the baseline CT scans within 1 week before chemotherapy and underwent the restaging CT examination within the 3-week interval between neoadjuvant chemotherapy and surgery. Scanning was performed by using Siemens Somatom Definition dual-source spiral CT (SOMATOM Definition, SOMATOM Definition Flash; Siemens Healthcare). Patients were entreated to fast from solid food for at least 6 h before CT examination, then drank 600–1000 ml water and were injected with 10 mg of anisodamine in order to make sure that the stomach wall was stretched. Also, the patients were asked to hold their breath during the scanning to prevent respiratory artifacts. All patients were in the supine position and the scanning range is from the phrenic tip to the lower edge of the symphysis pubis, covering the upper or the entire abdomen. Following the unenhanced scan, iodinated contrast agent (ioversol, 300 mgl/ml, GE Healthcare) was injected intravenously by using a high-pressure syringe (Ulrich, Medical). The infusion volume was 1.5 ml/kg and the flow rate of 3–4 ml/s. The arterial phase series was obtained with a post-injection delay of 30 s, and the venous phase with a post-injection delay of 60 s. The delay scan was conducted if necessary with a 180-s delay.

The parameters for abdomen CT were as follows: 120 kVp tube voltage, 230 mA tube current, 1/1.5 mm section thickness, 35–50 cm field of view, 512 × 512 matrix, 0.5 s rotation time, and 1.2 pitch. The value of the convolution kernel is B31f (SOMATOM Definition)/I30f (SOMATOM Definition Flash, using an iterative algorithm), and the collimation is 64 × 0.6mm/128 × 0.6mm. CT acquisition was performed as a spiral data set and the imaging review was with 1 or 1.5 mm contiguous axial reconstruction.

### CT-based radiomics analysis

#### Image segmentation

Radiologists (Q.M.X. and C.S.Z.) made independent image segmentation without accessing any clinical information of patients. One junior radiologist (Q.M.X.) manually segmented the tumor region on portal venous phase baseline and restaging CT imaging studies slice by slice as the region of interest (ROI) using the software for sophisticated image post-process (Dr. Wise ^TM^ Software http://label.deepwise.com/). One senior radiologist (C.S.Z.) reassessed and ensured the segmentation quality until reaching consensus.

#### Radiomics features extraction

We extracted radiomics features from tumor VOI using the python package pyradiomics (Version 2.2.0) [12]. We applied the Wavelet filter and Laplace of Gaussian filter [13] with different sigma values to the original CT images to enhance the discrimination of radiomics features. Specifically, Wavelet filter applied a high or a low pass filter in each dimension of signal; thus, we achieved 8 decompositions per level; Laplace of Gaussian filter emphasized coarser texture with higher sigma and finer texture with lower sigma. Then we resampled all the images with a new pixel spacing of 1.0 mm in all three dimensions, to exclude the disturbance caused by various scales by the interpolator of sitkBSpline in python package SimpleITK [14]. Finally, we extracted 1231 pre-defined features from both original and filtered images above, consisting of 6 classes, including first order statistics, shape-based, gray-level co-occurrence matrix (GLCM), gray-level run length matrix (GLRLM), gray-level size zone matrix (GLSZM), and gray-level dependence matrix (GLDM) (Table S2).

#### Model development

**Fig. 3:**
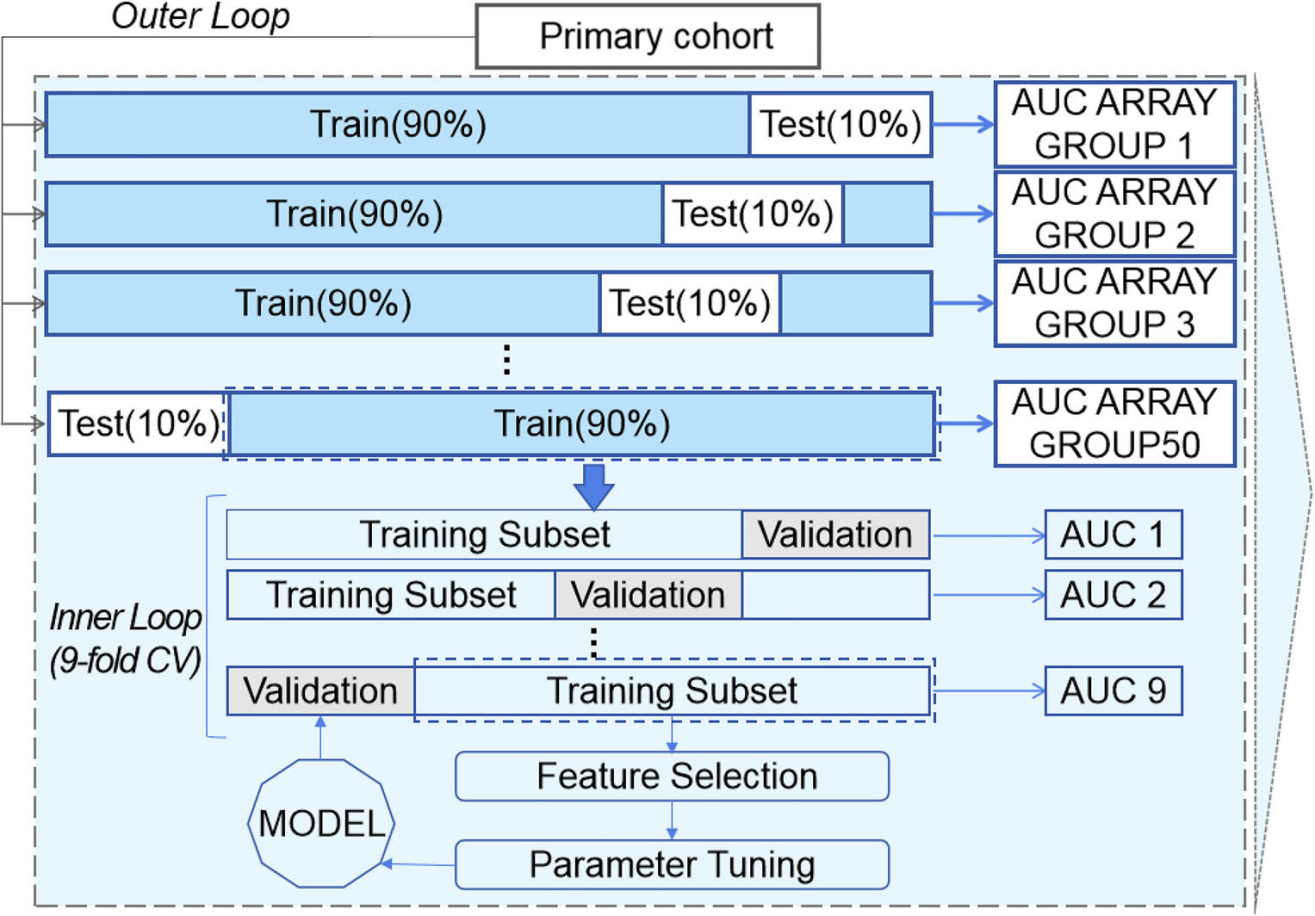
Illustration of the nested cross-validation structure. Note: AUC, area under the curve; CV, cross-validation

We developed four machine learning prediction models [15–18] and applied seven feature selection methods [19–25] for comparison in the primary cohort (*n* = 206). We used the nested cross-validation for both the PR model and DR model to find the best hyperparameters and optimize the number of features (Fig. 3) (Detail in Supplementary Appendix 1). Firstly, the whole dataset was divided randomly 50 times using stratified sampling in the outer loop, in which 10% of the dataset was used as a test set and the other 90% was used as a training set, forming 50 groups. In the inner loop, nine-fold cross-validation was applied to the training set to find the hyperparameters that help build the best model with highest average performance on the validation sets. Then this model was evaluated on the dependent test set in the outer loop to optimize the number of features. Therefore, we got an average score of 50 test sets and a model combining the prediction of all those 50 models built above in the end. Four classification models were tried in order to find the best model, including random forest, logistic regression, linear SVC, and K neighbors classifier. At the same time, feature selection methods were tried for model building, including *F*-test, mutual-information, recursive feature elimination, Pearson correlation coefficient, Wilcoxon rank-sum test, L1-based feature selection with linear SVC, L1-based feature selection with logistic regression. For each feature selection method above, different numbers of feby models were tried. The average area under the receiver operating characteristic curves (AUCs) of models on testing sets in nested CV structure was used to estimate models’ performance. Finally, we calculated the average of these two scores and got a merged result of the PR and DR model to improve the detection ability of models.

### Radiomics models’ validation and comparison with the clinical conventional method

Two testing cohorts were used for validation of each prediction task (Figs. 1 and 2). Testing cohort I (*n* = 40) was for assessing the evaluation performance of the radiomics model after 3–4 cycles’ chemotherapy. While testing cohort II (*n* = 46) was used for evaluating models’ ability of early detection of achieving pDS (Figure S2).

We made a comparison between the DR model and conventional RECIST method which is based on reduction of the diameter of tumor for evaluation of response to chemotherapy in both two external validation cohorts. The criterion of RECIST on CT images was stated in Supplementary Appendix 2.

### Protocols of treatment

The SEEOX regimen (*n* = 186, 63.7%) and SOX regimen (*n* = 106, 36.3%) were used for neoadjuvant chemotherapy in AGC patients. Both treatment courses consisted of 3 cycles (each, 2-week administration and 1-week withdrawal) and followed by surgery within 3 weeks. However, we found that 12 patients demanded one more cycle due to non-respond or other reasons. Also, we had 46 patients who took less than 3 cycles mainly because of intolerability (details of the treatment protocols were in Supplementary Appendix 3).

### Ascertainment of pDS

We separated patients into two clinical groups for ascertainment of pDS: pDS (Figure S3) and non-pDS (Figure S4) by comparing pre-chemotherapy cTNM stage (where c means clinical) [26, 27] and post-chemotherapy ypTNM stage (where y means after neoadjuvant therapy and p means pathologic stage) [28]. The two radiologists (Q.M.X. and C.S.Z.) evaluated the cTNM stage on baseline CT by consensus, and we obtained the ypTNM stage from surgical records. The pDS was defined as the tumor pathologically confirmed reducing stage after the neoadjuvant chemotherapy and the unresectable factor was removed, while non-pDS means the tumor stage was not changed, tumor progression, or remaining unresectable after the treatment. Supplementary Appendix 4 describes the criterion of evaluation of CT-based cTNM stage and resectability of tumor (Figure S5).

### Statistical analysis

We used SPSS v15.0 (SPSS Inc.) and MedCalc statistical software for statistical analysis. The AUC value and its 95% IC, sensitivity, specificity, and accuracy were listed to assess the model performance. We used the Delong test to calculate the 95% confidence interval (CI) for each AUC value and the net reclassification improvement (NRI) index between the radiomics model and clinical method. The receiver operating characteristic (ROC) curve showed the performance of models by potting the true positive rate (sensitivity) against the false positive rate (1-sensitivity). Chi-square test and Fisher’s exact test were to compare categorical data. A group difference was considered to be significant if the two-sided *p* value is less than 0.05.

## Results

### Patient characteristics

The cohort has 218 (74.7%) men and 74 (25.3%) women, with a median age of 61 years (interquartile range, 39.0–61.0 years). The median age among men was 61 years (interquartile range, 53.0–67.0 years) and the median age among women was 57 years (interquartile range, 47.8–64.0 years). No statistical difference in age was found between men and women in this cohort. Differentiation state was significantly associated with chemosensitivity (Table 1). Before neoadjuvant chemotherapy, 60 patients (20.5%) had clinical stage (cStage) II, 220 (75.4%) had cStage III, and 12 (4.1%) had cStage IV. After the treatment, 40 (14.7%) had ypT0N0-3M0 (ypTNM refers to the post-chemotherapy pathologic stage of tumor), 34 (11.6%) had ypStage I, 85 (29.1%) had ypStage II, 127 (43.5%) had ypStage III, and 8 (2.7%) had cStage IV (Table 2). In total, 108 patients (37.0%) had pDS (Figure S2) while 184 patients (63.0%) had non-pDS (Figure S3). No significant differences were among the three cohorts in chemotherapy response (*p* = .863). Among these patients, 47 were diagnosed as difficult to be resected (Figure S4) due to tumor infiltration of or lymph nodes fused and wrapped adjacent structures (left gastric artery, *n* = 27; hepatoduodenal ligament, *n* = 3; pancreas, *n* = 6; liver, *n* = 8; duodenum, *n* = 2; transverse colon, *n* = 1).

**Table 1:** Characteristics of patients in the primary cohort and two testing cohorts

Variables	Primary cohort (*n* = 206)			Testing cohort I (*n* = 40)			Testing cohort II (*n* = 46)		
	pDS	Non-pDS	*p* value	DS	Non-DS	*p* value	DS	Non-DS	*p* value
Gender			.723			.215			1.000
Male	59 (28.6)	96 (46.6)		9 (22.5)	18 (45.0)		12 (26.1)	24 (52.2)	
Female	18 ( 8.7)	33 (16.1)		7 (17.5)	6 (15.0)		3 (6.5)	7 (15.2)	
Age			.737			.272			.267
≥ 65	25 (12.1)	39 (18.9)		2 (5.0)	7 (17.5)		4 (8.7)	8 (17.4)	
< 65	52 (25.3)	90 (43.7)		14 (35.0)	17 (42.5)		11 (23.9)	23 (50.0)	
Primary tumor site			.773			.595			.794
Fundus	30 (14.6)	46 (22.3)		4 (10.0)	9 (22.5)		4 (8.7)	9 (19.6)	
Body	12 (5.8)	24 (10.7)		3 (7.5)	6 (15.0)		7 (15.2)	11 (23.9)	
Antrum	35 (17.0)	59 (28.5)		9 (22.5)	9 (22.5)		4 (8.7)	11 (23.9)	
Differentiation			.001			.048			.049
Well	7 (3.4)	2 (0.1)		1 (2.5)	0 (0.0)		2 (4.3)	1 (0.2)	
Moderately	30 (14.6)	32 (15.5)		7 (17.5)	6 (15)		5 (10.9)	5 (10.9)	
Poorly	39 (18.9)	96 (46.5)		6 (15.0)	20 (50.0)		6 (13.0)	27 (58.7)	
Chemotherapy			.856			.332			.249
SEEOX	57 (27.7)	94 (45.6)		11 (27.5)	12 (30.0)		7 (15.2)	20 (43.5)	
SOX	20 (9.7)	35 (17.0)		5 (12.5)	12 (30.0)		8 (17.4)	11 (23.9)	
Gastrectomy*			.232			.062			.438
PG	4 (2.1)	1 (0.5)		1 (2.7)	0 (0.0)		3 (6.5)	2 (4.4)	
TG	47 (24.6)	70 (36.6)		6 (16.2)	15 (40.5)		11 (23.9)	26 (56.5)	
DG	26 (13.6)	43 (22.5)		9 (24.3)	6 (16.2)		1 (2.2)	3 (6.5)	

Note: Data are numerators, with percentages in parentheses. *p* value is derived from the univariable association analyses between each of the clinical characteristic variables and treatment response after neoadjuvant chemotherapy. *pDS*, pathological downstaging; *SEEOX*, oxaliplatin, etoposide, epirubicin, and S-1; *SOX*, s1 and oxaliplatin; *PG*, proximal gastrectomy; *TG*, total gastrectomy; *DG*, distal gastrectomy. *Patients who did not receive radical surgery were not included

**Table 2:** The TNM stage of patients before and after neoadjuvant chemotherapy

	Primary cohort	Testing cohort I	Testing cohort II
cTNM stage			
cT3N0M0	23 (11.2)	2 (5.0)	1 (2.2)
cT3N+M0	58 (28.2)	11 (27.5)	7 (15.2)
cT4aN0M0	28 (13.6)	4 (10.0)	2 (4.3)
cT4aN+M0	90 (43.7)	20 (50.0)	34 (73.9)
cT4bN+M0	7 (3.3)	3 (7.5)	2 (4.3)
ypTNM stage			
ypT0N0M0	19 (9.2)	4 (10.0)	1 (2.2)
ypT0N1M0	10 (4.9)	1 (2.5)	0 (0.0)
ypT0N2M0	1 (0.5)	1 (2.5)	0 (0.0)
ypT0N3M0	2 (1.0)	1 (2.5)	0 (0.0)
ypT1N0M0	10 (4.9)	0 (0.0)	4 (8.7)
ypT1N1M0	1 (0.5)	0 (0.0)	0 (0.0)
ypT1N2M0	2 (1.0)	2 (1.0)	0 (0.0)
ypT1N3M0	1 (0.5)	0 (0.0)	1 (2.2)
ypT2N0M0	10 (4.9)	4 (1.9)	5 (10.9)
ypT2N1M0	7 (3.4)	1 (0.5)	1 (2.2)
ypT2N2M0	10 (4.9)	2 (1.0)	3 (6.5)
ypT2N3M0	4 (1.9)	0 (0.0)	0 (0.0)
ypT3N2M0	5 (2.4)	0 (0.0)	0 (0.0)
ypT4aN0M0	38 (18.4)	5 (2.4)	8 (17.4)
ypT4aN1M0	25 (12.1)	3 (1.4)	7 (15.2)
ypT4aN2M0	23 (11.2)	6 (2.9)	7 (15.2)
ypT4aN3M0	29 (14.1)	6 (2.9)	8 (17.4)
ypT4b	4 (1.9)	1 (0.4)	1 (2.2)
M1 post-treatment	5 (2.4)	3 (1.5)	0 (0.0)

Note: Data are numerators, with percentages in parentheses. The cTNM stage refers to the pre-chemotherapy clinical stage of tumor; the ypTNM stage refers to the post-chemotherapy pathologic stage of tumor

### Performance of radiomics models

We systematically examined twenty-eight combinations of feature selection and classification methods based on restaging CT in the primary cohort (161 restaging CECT scans) (Figure S6). Table S3 showed superior prediction outcomes with mean AUC ranged from 0.765 to 0.919. We found that there were three cross-combination machine learning methods achieving high AUC (> 0.900), of which were (a) the feature selection method of Wilcoxon and classifier of linear SVC, (b) the feature selection method of *F*-test and classifier of linear SVC, and (c) the feature selection method of *F*-test and classifier of logistic regression. Among these three models, the optimal model consisted of the feature selector of Wilcoxon and classifier of linear SVC.

Based on this optimal combination, our PR radiomics model (trained on 159 baseline CECT) was predictive (AUC 0.779, 95% CI: 0.774, 0.784) using 67 radiomics features (Figure S5a). Also, our DR model (trained on 161 restaging CECT) showed a promising result (AUC 0.919, 95% CI: 0.900, 0.939) using 92 radiomics features (Figure S5b).

### Radiomics models’ validation and comparison with the clinical method

**Fig. 4:**
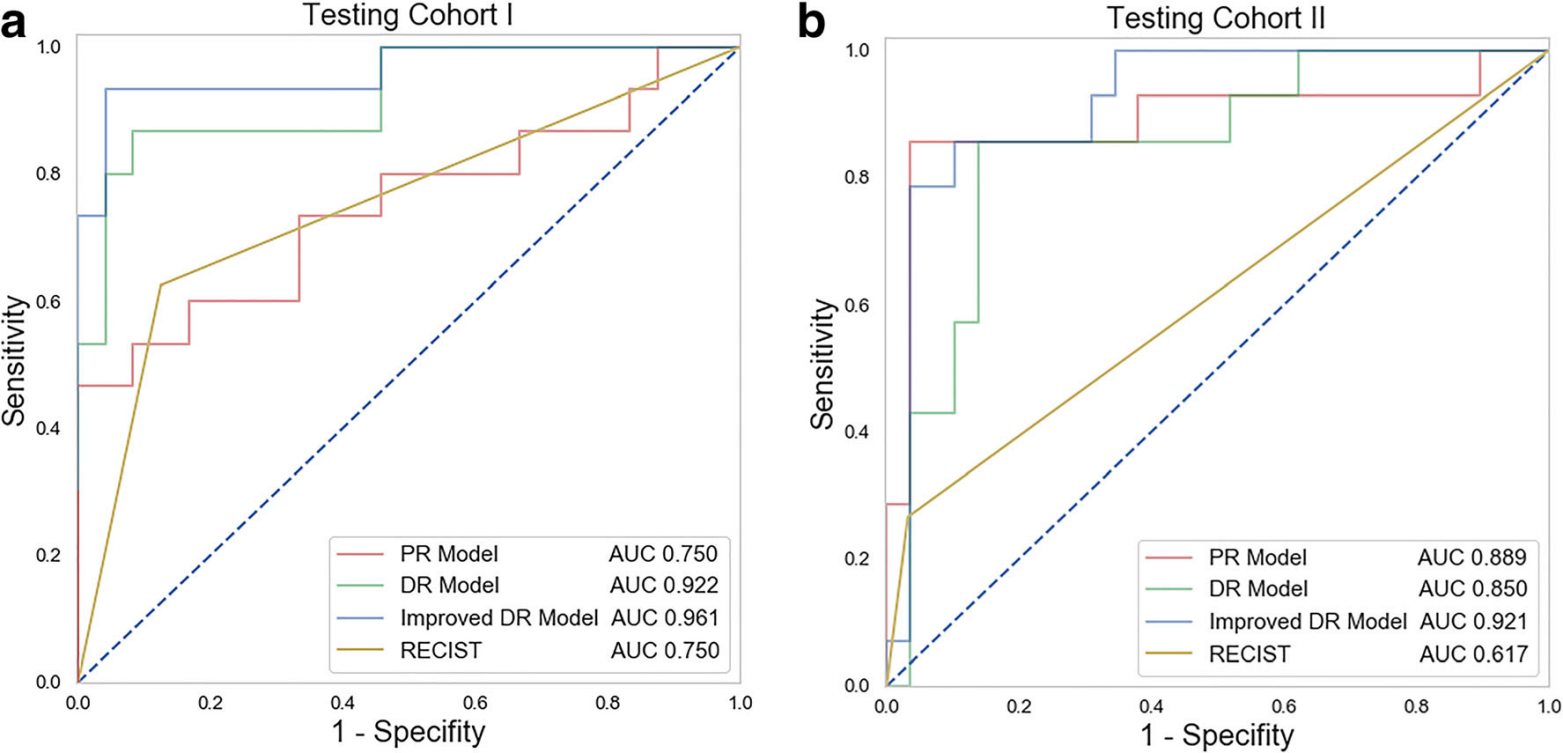
Receiver operating characteristic curves for the prediction, detection, the improved detection radiomics models, and the RECIST method in (**a**) testing cohort I and (**b**) testing cohort II, respectively. Note: RECIST, Response Evaluation Criteria in Solid Tumors

**Fig. 5:**
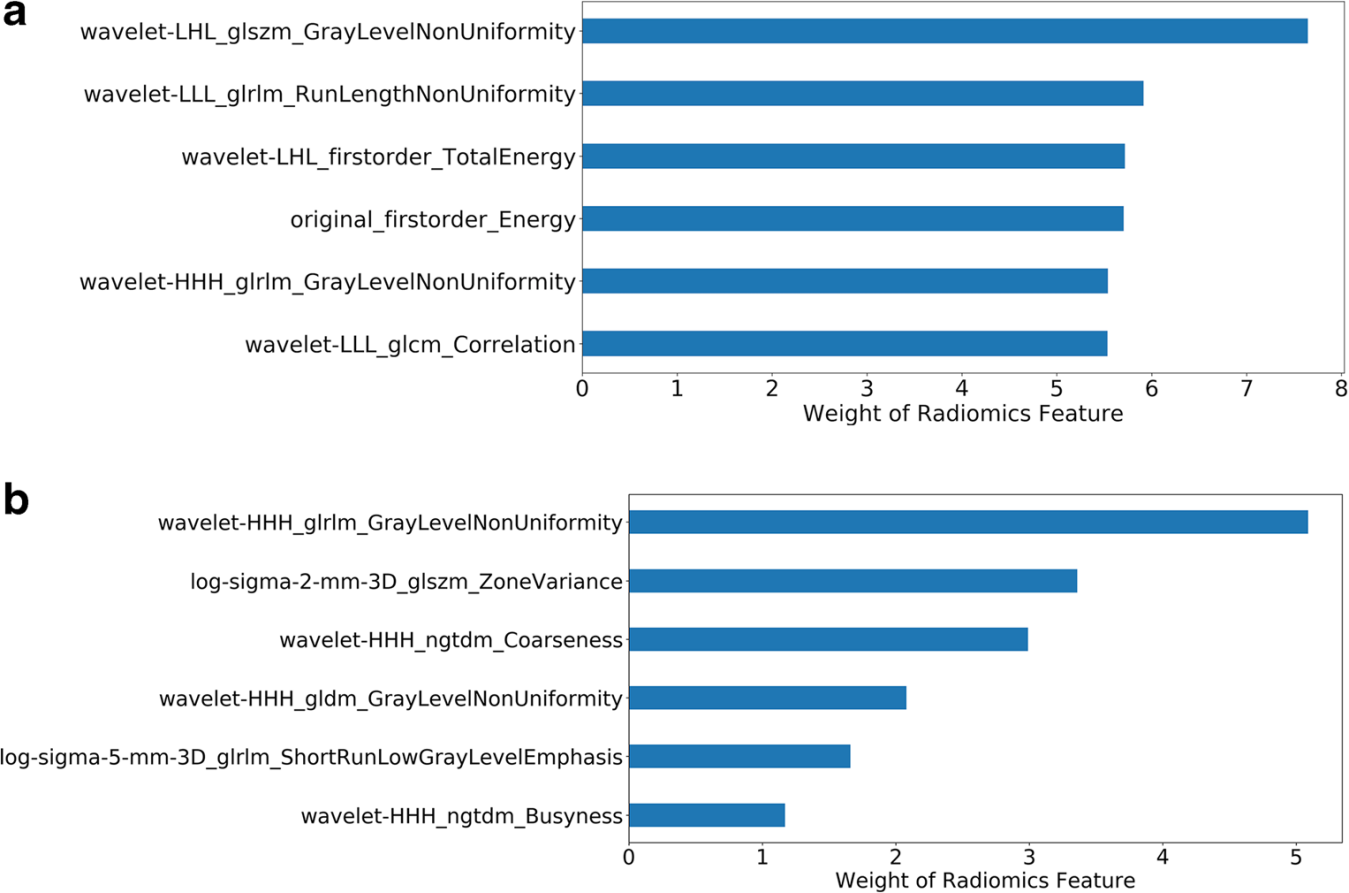
Radiomics features with the top six weights in (**a**) the prediction and (**b**) detection radiomics model, respectively

Our PR model presented a good predictive ability of achieving pDS before therapy on testing cohort I (AUC 0.750, ACC 0.769) and testing cohort II (AUC 0.889, ACC 0.837) (39 and 43 baseline CECT, respectively) with high specificity (0.958 and 0.966) (Table 3). The DR model also had a good diagnostic value (AUC 0.922, ACC 0.897, and AUC 0.850, ACC 0.860) of pDS in both testing cohorts (40 and 46 restaging CT, respectively) (Table 3). This model outperformed the clinical RECIST method (NRI = 39.5%, Z = 2.04, *p* < .05 and NRI = 35.4%, Z = 1.63, *p* < 0.05). Specifically, this DR radiomics model was more accurate and sensitive than the clinical method using RECIST (ACC 0.897 and 0.775, SEN 0.867 and 0.625) when diagnosing pDS after the treatment in testing cohort I. Meanwhile, the DR model also performed well for early detection of pDS in testing cohort II, while the RECIST showed no diagnostic value (AUC 0.617, 95% CI: 0.462–0.757, *p* = 0.0557). Furthermore, the discrimination of pDS was increased in the improved DR model using the average scores of outcomes of PR and DR models in both testing cohorts (AUC 0.961, ACC 0.897, and AUC 0.921, ACC 0.907). Figure 4 shows the four receiver operating characteristic (ROC) curves for PR, DR, improved DR models, and RECIST method. Also Fig. 5 lists the radiomics features with the top six weights in PR and DR models. Radiomics calculation formulas were listed in Supplementary Appendix 5.

**Table 3:** Performance of the PR, DR, and improved DR models in two testing cohorts

	Testing cohort I				Testing cohort II			
	PR	RESCIT	DR	Improved DR	PR	RECIST	DR	Improved DR
AUC	0.750	0.750	0.922	0.961	0.889	0.617	0.850	0.921
	(0.579–0.921)	(0.588–0.873)	(0.799–0.985)	(0.898–1)	(0.756–1)	(0.462–0.757)	(0.799–0.985)	(0.835–1)
*p* value	0.009*	0.001*	0.000*	0.000*	0.000*	0.056	0.000*	0.000*
SEN	0.467	0.625	0.867	0.733	0.571	0.266	0.857	0.786
SPE	0.958	0.875	0.917	1	0.966	0.968	0.862	0.966
ACC	0.769	0.775	0.897	0.900	0.837	0.739	0.860	0.907

Note: *p value < .05. *PR*, prediction radiomics; *DR*, detection radiomics; *AUC*, area under the curve; *SEN*, sensitivity; *ACC*, accuracy

## Discussion

Our prediction radiomics (PR) model and detection radiomics (DR) model using baseline and restaging contrast-enhanced CT (CECT) performed strongly on prediction and early detection of pathological downstaging (pDS) for advanced gastric cancer (AGC). The two models revealed potential risk factors and current benefits of neoadjuvant chemotherapy, which can potentially assist surgical decision-making.

Our PR model performs patient stratification on admission to reduce additional toxicity of inefficient treatment for non-respond patients [2, 3]. Our improved DR radiomics model provides early detection of response after each cycle of the chemotherapy course, allowing timely surgical decision for those responsive candidates who achieve pDS. Such model preoperatively distinguishes non-responders after complete course of chemotherapy to avoid improper surgical treatment. Notably, our models made correct predictions for (a) patients (pDS *n* = 9, non-pDS *n* = 3) who had initially deemed irrectable tumors; (b) patients (pDS *n* = 4) that ceased chemotherapy at halfway due to toxicity and underwent early trials of dissection, and were found already achieved pDS; and (c) patients (non-pDS *n* = 3) confirmed to have inoperable disease at improper surgery, a known trigger for metastasis requiring palliative management [29]. In our radiomics findings, wavelet-based and energy-based features achieved the highest feature weights as confirmed in previous reports [15, 30, 31]. These features provide detailed information on sight histologic changes in tumor [7, 8] including decreased tumor cell density, fibrosis, mucus lakes, and chronic inflammatory infiltrates [32]. Furthermore, our DR model outperformed the CT-based Response Evaluation Criteria in Solid Tumors (RECIST) method, which is in line with the commonly used endoscopic assessment for AGC patients [33]. This indicated that the CT radiomics may potentially surpass endoscopy or CT gastrography as a useful method for response evaluation of AGC.

Our study has limitations. First, our cohort contained fewer patients achieving pDS (58.7%); the imbalance of groups may influence the performance of predictive models. Second, images with low-quality scores were excluded by the evaluation process of CT images quality, which might result in potential image selection bias. Third, despite the examination of CT images analyzed by consensus between two reviewers, inter-variability still existed that requires additional assessment. Finally, although testing cohorts were conducted to improve reliability, our research is based on retrospective analysis and further prospective multi-center studies with more cases stratified would be helpful to validate our findings.

In conclusion, we demonstrated that CT-based radiomics models using baseline and restaging CECT offer predictive value for pDS before, during, and at the end of neoadjuvant chemotherapy, which can potentially support clinical decision-making for AGC patients. Future studies will be warranted to explore the generalized utility of our models and translate our findings into clinical practice.

## Supplementary Information

ESM 1 (DOCX 3571 kb)

## Abbreviations

AGC: Advanced gastric cancer
AUC: Area under the curve
CECT: Contrast-enhanced CT
CT: Computed tomography
CV: Cross-validation
DG: Distal gastrectomy
DR: Detection radiomics model
GC: Gastric cancer
GLCM: Gray-level co-occurrence matrix
GLDM: Gray-level dependence matrix
GLRLM: Gray-level run length matrix
GLSZM: Gray-level size zone matrix
pDS: Pathological downstaging
PG: Proximal gastrectomy
PR: Prediction radiomics model
RECIST: Response evaluation criteria in solid tumors
ROC: Receiver operating characteristic
ROI: Region of interest
TG: Total gastrectomy
VOI: Volume of interest

## Declarations

### Guarantor

The scientific guarantor of this publication is Guangming Lu.

### Statistics and biometry

No complex statistical methods were necessary for this paper.

### Methodology

• retrospective

• diagnostic or prognostic study

• performed at one institution
