# Supplementary material for: Advanced gastric cancer: CT radiomics prediction and early detection of downstaging with neoadjuvant chemotherapy
Source: Eur Radiol. 2021 Apr 28;31(11):8765–74. doi: 10.1007/s00330-021-07962-2 (PMC8523390; doi:10.1007/s00330-021-07962-2)

**Appendix 1 Feature selection in model development**

We developed four machine-learning prediction models for comparison, including random forest [17], logistic regression [18], Linear SVC [19] and K Neighbors Classifier [20]. At the same time, several feature selection methods were used for model building, including F-test [21], mutual-information [22], recursive feature elimination [23], Pearson correlation coefficient [24], Wilcoxon rank-sum test [25], L1-based feature selection with Linear SVC [26], L1-based feature selection with Logistic Regression [27].

During the training and internal validation, we first divided the whole dataset randomly 50 times using stratified sampling in the outer loop (in which 10 percent of the dataset was used as a test set and the other 90 percent was used as a training set) forming 50 groups. We evaluated the selected model on the dependent test set in the outer loop to optimize the number of features. For each radiomic model, different numbers of features (range from 5 to 200) used by models were attempted (Figure S2). The total number of features including duplicate cases involved in model development was 50 × n (n represented number of features). All features involved in model training were used in radiomics score calculation formula and the average weights of these features were calculated as the radiology score calculation weights. In the inner loop, we applied nine-fold cross-validation to the training set to find the hyper-parameters that help build the best model with the highest average performance for the validation. Therefore, we got an average score of 50 test sets and a model combining the prediction of all those 50 models built above for each model. The average area under the receiver operating characteristic curves (AUCs) of models on testing sets in nested CV structure was used to estimate models’ performance. Finally, we calculated the average of these two scores and got a merged result of the PR and DR model to improve the detection ability of models.

In our experiment, the optimum number of features used in the PR and DR model development were 9 and 79 respectively, the total number of features used in radiomics score calculation formula were 67 and 92 respectively.

**Appendix 2 The criterion of RECIST on CT images**

We made a comparison between the radiomics model and the conventional RECIST method in both two testing cohorts. The axial baseline CT and restaging CT images in the testing cohort 1 (n = 40) and testing cohort 2 (n = 46) were reviewed by the two radiologists and measured by one of the radiologists (Qinmei Xu) followed the Response Evaluation Criteria in Solid Tumors (RECIST1.1) guideline. We defined responsive outcomes as tumor downstaging after chemotherapy which includes the disappearance of all target lesions or at least a 30% decrease in the sum of diameters of target lesions as well as any pathological lymph nodes must have reduction in short axis to < 10 mm. While Non-responsive outcome was shown at least a 20% increase in the sum of diameters of target lesions, the sum must also demonstrate an absolute increase of at least 5 mm; or the appearance of one or more new lesions is also considered progression; any other circumstances.

**Appendix 3 Treatment protocols**

Two types of treatment regimens were used in our study. (1) SEEOX regimen (n = 159, 67.1%) via combined intra-arterial and intravenous administration: Intra-arterial administration of oxaliplatin (100 mg/m^2^), etoposide (80 mg/m^2^) and epirubicin (30 mg/m^2^) by Seldinger method on day 1 and oral S-1(120mg) on days 1-14. (2) SOX regimen (n = 78, 32.9%) via intravenous administration: oxaliplatin 130 mg/m^2^ for intravenous injection on day 1; oral S-1(120 mg) on days 1-14. Both treatment schedules consisted of 3 cycles (each, 2-week administration and 1-week withdrawal) and followed by surgery within three weeks. These treatment regimens have not changed since patient recruitment was completed.

**Appendix 4 CT evaluation of the tumor’s TNM stage and resectability**

The cTNM stage before chemotherapy was evaluated on the baseline CT images by the two radiologists (Qinmei Xu and Changsheng Zhou). The radiologists were aware that the patients were diagnosed with GC and treated with chemotherapy before surgery, but they were blinded to endoscopic or histopathologic results of patients. The majority of the baseline CT scans (270 of 289; 93.4%) and the restaging CT scans (284 of 289; 98.2%) were performed at our institution. We diagnosed the clinical T stage (depth of invasion: cT3/cT4a/T4b) and N stage (lymph nodes status: cN0/cN+) based on these CT scans. The multi-detector row CT criteria currently used to diagnose T3 gastric cancer are “tumors penetrating the muscularis propria and extending into the subperitoneal spaces of the greater and lesser omentum but without violation of the serosa (the visceral peritoneum) covering these structures”. “A nodular or irregular outer gastric wall layer with the existence of the perigastric fat” should be staged T4a. T4b, infiltration of adjacent organs, perigastric fat are not visible. The cN+ (malignant lymph nodes) were classified as those with short-axis diameters ≥ 10 mm for perigastric lymph nodes on CT scans.

The ypTNM stage (where y means after neoadjuvant therapy and p means pathologic stage, according to the American Joint Committee on Cancer 8th edition) after neo-adjuvant chemotherapy was obtained from the histopathologic report of the surgical resection specimens of those patients who underwent gastrectomy. We used the clinical stage on the CT report after treatment as the post-therapy stage for patients without radical resection due to distant metastases.

In order to evaluate the ability of the model performance in early detection of unresectable tumor downstaging during the period of chemotherapy, a tumor was also designated as being either resectable or unresectable on baseline CT images by two radiologists (Chen Ye and Qinmei Xu, with 3 years experience in diagnosis and treatment of GC and 4 years experience in gastric CT imaging, respectively). Multi-detector row CT findings indicated locally AGC is difficult to be resected, including infiltration of adjacent structures (left gastric artery, hepato-duodenal ligament, pancreas, liver, duodenum, etc.), fusion of the lymph nodes adjacent to the main blood vessels or wrapping the roots of the blood vessels, and distant metastatic (peritoneal metastasis, liver metastasis, etc.).

**Appendix 5 Radiomics score calculation formula**

**1. The portal venous phase baseline CT images based radiomics score was calculated for each patient based on the features as follows:**

Score = - 19.2056 + 7.6461 × wavelet-LHL_glszm_GrayLevelNonUniformity + 5.914 × wavelet-LLL_glrlm_RunLengthNonUniformity + 5.717 × wavelet-LHL_firstorder_TotalEnergy + 5.7043 × original_firstorder_Energy + 5.5387 × wavelet-HHH_glrlm_GrayLevelNonUniformity + 5.5334 × wavelet-LLL_glcm_Correlation - 4.7657 × wavelet-HLL_gldm_GrayLevelNonUniformity + 4.5879 × wavelet-LHL_glszm_ZoneVariance - 4.487 × log-sigma-3-mm-3D_firstorder_TotalEnergy - 4.4867 × wavelet-HLH_gldm_DependenceNonUniformity - 4.4298 × wavelet-LLH_gldm_GrayLevelNonUniformity + 4.2975 × wavelet-LLL_gldm_DependenceNonUniformity + 4.2823 × wavelet-LHH_firstorder_Energy + 3.9928 × wavelet-LLH_firstorder_Energy - 3.9279 × log-sigma-1-mm-3D_firstorder_TotalEnergy + 3.6164 × wavelet-HLH_firstorder_TotalEnergy + 3.5893 × wavelet-LHL_gldm_DependenceNonUniformity - 3.5474 × general_info_VoxelNum + 3.536 × original_shape_SurfaceVolumeRatio - 3.4714 × wavelet-HHH_gldm_DependenceNonUniformity + 3.4026 × log-sigma-1-mm-3D_gldm_GrayLevelNonUniformity + 3.2307 × wavelet-LHL_glrlm_GrayLevelNonUniformity + 3.1024 × original_glszm_ZoneVariance + 3.1014 × log-sigma-1-mm-3D_glszm_LargeAreaEmphasis - 3.0276 × wavelet-HLL_firstorder_TotalEnergy + 2.8206 × wavelet-LHL_gldm_GrayLevelNonUniformity - 2.7988 × wavelet-LHL_firstorder_Energy - 2.795 × wavelet-HLH_gldm_GrayLevelNonUniformity - 2.7445 × wavelet-LHH_glrlm_GrayLevelNonUniformity + 2.6912 × wavelet-LLH_firstorder_TotalEnergy - 2.6791 × wavelet-HHH_gldm_GrayLevelNonUniformity + 2.6379 × log-sigma-4-mm-3D_firstorder_Energy + 2.595 × wavelet-LHH_gldm_GrayLevelNonUniformity + 2.5632 × log-sigma-4-mm-3D_gldm_DependenceNonUniformity + 2.5419 × wavelet-LHL_glrlm_RunLengthNonUniformity + 2.4793 × wavelet-LLL_glcm_Imc1 + 2.4355 × original_glszm_LargeAreaEmphasis - 2.3145 × wavelet-LHH_glszm_ZoneVariance + 2.2678 × wavelet-HHH_glszm_LargeAreaHighGrayLevelEmphasis + 2.2 × wavelet-LLL_firstorder_Energy - 2.1975 × wavelet-HHH_firstorder_TotalEnergy - 2.1872 × wavelet-HHL_gldm_GrayLevelNonUniformity - 2.005 × wavelet-LHH_firstorder_TotalEnergy - 1.9949 × log-sigma-3-mm-3D_firstorder_Energy - 1.9937 × wavelet-HLH_glszm_LargeAreaEmphasis - 1.9592 × log-sigma-5-mm-3D_firstorder_TotalEnergy - 1.9008 × wavelet-HHL_firstorder_Energy + 1.8381 × wavelet-HLH_firstorder_Energy + 1.8224 × log-sigma-2-mm-3D_firstorder_TotalEnergy - 1.774 × wavelet-HLH_glszm_ZoneVariance + 1.7474 × wavelet-LLL_firstorder_TotalEnergy + 1.659 × wavelet-HLH_glrlm_GrayLevelNonUniformity + 1.6025 × wavelet-LHL_glszm_LargeAreaEmphasis + 1.5247 × log-sigma-2-mm-3D_firstorder_Energy - 1.2789 × original_firstorder_TotalEnergy + 1.2219 × wavelet-LHH_glszm_ZonePercentage + 1.081 × wavelet-HHL_glszm_ZoneVariance + 1.0787 × log-sigma-4-mm-3D_firstorder_TotalEnergy + 0.9676 × wavelet-LLL_gldm_DependenceEntropy - 0.9613 × wavelet-HLL_glrlm_GrayLevelNonUniformity - 0.96 × original_shape_Volume + 0.9175 × wavelet-HHH_glszm_LargeAreaEmphasis + 0.8826 × wavelet-LLL_glszm_ZoneEntropy + 0.8514 × wavelet-HLL_glszm_LargeAreaEmphasis - 0.849 × wavelet-HHH_glszm_ZoneVariance - 0.8308 × wavelet-LLL_glcm_Imc2 - 0.8303 × wavelet-LLH_glszm_ZoneVariance + 0.7514 × log-sigma-1-mm-3D_glszm_ZoneVariance + 0.6834 × wavelet-LHH_gldm_DependenceNonUniformity - 0.5924 × wavelet-LLH_glszm_LargeAreaEmphasis - 0.59 × wavelet-HLL_glszm_ZoneVariance - 0.5781 × wavelet-LHH_glszm_LargeAreaEmphasis - 0.5768 × log-sigma-1-mm-3D_firstorder_Energy + 0.5714 × log-sigma-1-mm-3D_glrlm_GrayLevelNonUniformity + 0.5686 × wavelet-HHL_glszm_LargeAreaEmphasis + 0.4175 × wavelet-LLH_glrlm_GrayLevelNonUniformity + 0.4052 × wavelet-HHL_gldm_DependenceNonUniformity + 0.3897 × log-sigma-1-mm-3D_gldm_DependenceNonUniformity + 0.3365 × log-sigma-3-mm-3D_gldm_DependenceNonUniformity + 0.2816 × wavelet-LHH_glszm_LargeAreaHighGrayLevelEmphasis + 0.2534 × wavelet-HLH_glszm_LargeAreaHighGrayLevelEmphasis + 0.246 × log-sigma-2-mm-3D_gldm_DependenceNonUniformity - 0.1776 × log-sigma-5-mm-3D_gldm_DependenceNonUniformity + 0.1693 × wavelet-HHL_firstorder_TotalEnergy - 0.1294 × log-sigma-5-mm-3D_firstorder_Energy + 0.1098 × original_gldm_GrayLevelNonUniformity + 0.1067 × wavelet-HHL_glrlm_GrayLevelNonUniformity - 0.0592 × wavelet-HHH_firstorder_Energy - 0.0341 × wavelet-HLL_firstorder_Energy - 0.0261 × wavelet-HHL_glszm_LargeAreaLowGrayLevelEmphasis + 0.0058 × wavelet-HLH_gldm_DependenceNonUniformityNormalized + 0.0058 × wavelet-HHH_glszm_LargeAreaLowGrayLevelEmphasis

**2. The portal venous phase restaging CT images based radiomics score was calculated for each patient based on the features as follows:**

Score = 3.7761 + 5.0916 × wavelet-HHH_glrlm_GrayLevelNonUniformity + 3.3594 × log-sigma-2-mm-3D_glszm_ZoneVariance + 2.99 × wavelet-HHH_ngtdm_Coarseness + 2.1447 × wavelet-HLL_ngtdm_Coarseness + 2.0788 × wavelet-HHH_gldm_GrayLevelNonUniformity + 2.0288 × log-sigma-4-mm-3D_glszm_ZoneVariance + 1.6609 × log-sigma-5-mm-3D_glrlm_ShortRunLowGrayLevelEmphasis + 1.166 × wavelet-HHH_ngtdm_Busyness + 1.0781 × wavelet-HHH_gldm_DependenceNonUniformity + 1.0612 × wavelet-HLH_glrlm_GrayLevelNonUniformity + 0.9347 × log-sigma-2-mm-3D_glszm_LargeAreaEmphasis - 0.9029 × log-sigma-4-mm-3D_glszm_LargeAreaEmphasis + 0.8622 × wavelet-HLL_glszm_GrayLevelNonUniformity + 0.7892 × log-sigma-2-mm-3D_firstorder_TotalEnergy + 0.7458 × log-sigma-3-mm-3D_gldm_GrayLevelNonUniformity + 0.6705 × wavelet-LLH_gldm_GrayLevelNonUniformity + 0.6644 × log-sigma-5-mm-3D_gldm_LowGrayLevelEmphasis + 0.6424 × wavelet-LHH_gldm_DependenceNonUniformity + 0.6269 × wavelet-LLH_ngtdm_Coarseness + 0.6242 × wavelet-HHL_gldm_GrayLevelNonUniformity + 0.5654 × wavelet-HLH_gldm_DependenceNonUniformity + 0.4935 × original_shape_MeshVolume + 0.4397 × wavelet-HLH_ngtdm_Coarseness - 0.4099 × wavelet-HHH_firstorder_Energy + 0.4041 × wavelet-HHL_glszm_ZoneVariance + 0.3614 × wavelet-HLH_glszm_LargeAreaEmphasis + 0.358 × wavelet-HHL_glszm_LargeAreaEmphasis - 0.3493 × wavelet-LHH_glszm_SizeZoneNonUniformity + 0.328 × wavelet-LLL_firstorder_Energy + 0.3265 × log-sigma-3-mm-3D_glszm_LargeAreaEmphasis - 0.2502 × log-sigma-2-mm-3D_gldm_GrayLevelNonUniformity - 0.2417 × wavelet-HLL_gldm_GrayLevelNonUniformity - 0.2372 × wavelet-LHL_glszm_ZoneVariance + 0.2353 × wavelet-LHL_gldm_GrayLevelNonUniformity + 0.1941 × wavelet-

LHH_firstorder_TotalEnergy - 0.194 × wavelet-LLH_gldm_LargeDependenceHighGrayLevelEmphasis + 0.1655 × wavelet-HLL_glszm_ZoneVariance - 0.1568 × log-sigma-3-mm-3D_firstorder_TotalEnergy - 0.1526 × wavelet-LHL_ngtdm_Coarseness - 0.151 × wavelet-LHL_glszm_LargeAreaEmphasis + 0.1372 × wavelet-HHL_glrlm_GrayLevelNonUniformity + 0.1367 × log-sigma-3-mm-3D_glrlm_GrayLevelNonUniformity - 0.1354 × wavelet-LLL_firstorder_TotalEnergy - 0.1301 × wavelet-HHH_firstorder_TotalEnergy - 0.1251 × log-sigma-5-mm-3D_glrlm_LowGrayLevelRunEmphasis + 0.1247 × log-sigma-3-mm-3D_firstorder_Energy + 0.1053 × wavelet-HLL_glszm_SizeZoneNonUniformityNormalized - 0.1052 × wavelet-LLL_ngtdm_Contrast - 0.1047 × wavelet-LLH_glszm_ZoneVariance + 0.1013 × wavelet-HHL_firstorder_TotalEnergy + 0.1008 × original_shape_VoxelVolume + 0.0886 × wavelet-HHH_glrlm_RunLengthNonUniformity + 0.0858 × wavelet-LHL_firstorder_Energy - 0.0843 × log-sigma-3-mm-3D_glszm_ZoneVariance - 0.0821 × wavelet-HLH_glszm_ZoneVariance + 0.079 × wavelet-LHH_firstorder_Energy - 0.0783 × wavelet-LLL_glrlm_LongRunLowGrayLevelEmphasis + 0.0623 × log-sigma-4-mm-3D_firstorder_Energy + 0.0623 × log-sigma-4-mm-3D_firstorder_TotalEnergy - 0.0482 × wavelet-LLH_glszm_LargeAreaEmphasis + 0.0473 × wavelet-HHL_glrlm_ShortRunHighGrayLevelEmphasis + 0.0355 × wavelet-HHL_firstorder_Energy + 0.0355 × wavelet-HLH_firstorder_Energy + 0.0355 × wavelet-HLH_firstorder_TotalEnergy - 0.0168 × log-sigma-2-mm-3D_firstorder_Energy + 0.0124 × wavelet-HLL_glszm_SizeZoneNonUniformity + 0.0069 × log-sigma-5-mm-3D_gldm_LargeDependenceLowGrayLevelEmphasis

**Appendix 6 Table captions**

**Table S1**. **Evaluation Criterion of Baseline and Restaging CT Image.**

|  | Grade |  | Patients Number |
| --- | --- | --- | --- |
| Images  Artifacts | 0 | Serious artifacts such as motion artifacts which makes the image to have no diagnostic value. | 0 |
|  | 1 | Unclear outline of the lesion due to some artifacts which may affect the diagnosis result. | 46 |
|  | 2 | Some slight artifacts exist but do not affect the diagnosis. | 37 |
|  | 3 | No artifacts are found. The outline of the lesion is clear. | 405 |
| Gastric Filling | 0 | No media filling in the gastric. | 5 |
|  | 1 | Few media filling in the gastric. | 34 |
|  | 2 | The gastric is of good dilation. The mucosa is still a little wrinkled but can be clearly identified. | 79 |
|  | 3 | The gastric cavity is fully dilated and the mucosa is fully expanded. | 370 |

**Table S2**. **Number of Features Extracted from the CT Scans, Consisting of Six Classes.**

| Method | Number of features |
| --- | --- |
| First Order Statistics | 252 |
| Shape-based | 13 |
| Gray Level Cooccurrence Matrix (GLCM) | 322 |
| Gray Level Run Length Matrix (GLRLM) | 224 |
| Gray Level Dependence Matrix (GLDM) | 196 |
| Gray Level Size Zone Matrix (GLSZM) | 224 |

**Table S3**. **Mean AUC, Sensitivity, Specificity, and Accuracy of Twenty-eight Cross-combination Machine-learning Models**

| Radiomics models | AUC (95% CI) | Sensitivity | Specificity | Accuracy |
| --- | --- | --- | --- | --- |
| KNC_FSCR | 0.821 (0.792-0.851) | 0.604 | 0.834 | 0.748 |
| KNC_L1LR | 0.829 (0.802-0.855) | 0.653 | 0.820 | 0.757 |
| KNC_L1SVC | 0.809 (0.780-0.838) | 0.604 | 0.818 | 0.737 |
| KNC_MIFS | 0.852 (0.825-0.878) | 0.643 | 0.826 | 0.782 |
| KNC_PSCR | 0.765 (0.741-0.789) | 0.472 | 0.862 | 0.714 |
| KNC_RECS | 0.806 (0.777-0.835) | 0.594 | 0.838 | 0.746 |
| KNC_WLCX | 0.867 (0.842-0.891) | 0.613 | 0.872 | 0.774 |
| LSVC_FSCR | 0.901 (0.881-0.922) | 0.833 | 0.794 | 0.809 |
| LSVC_L1LR | 0.869 (0.841-0.897) | 0.788 | 0.778 | 0.781 |
| LSVC_L1SVC | 0.854 (0.827-0.880) | 0.784 | 0.792 | 0.789 |
| LSVC_MIFS | 0.881 (0.858-0.904) | 0.844 | 0.812 | 0.824 |
| LSVC_PSCR | 0.894 (0.874-0.914) | 0.823 | 0.758 | 0.783 |
| LSVC_RECS | 0.842 (0.814-0.870) | 0.780 | 0.746 | 0.759 |
| LSVC_WLCX | 0.919 (0.900-0.939) | 0.838 | 0.796 | 0.812 |
| LR_FSCR | 0.907 (0.889-0.929) | 0.797 | 0.858 | 0.835 |
| LR_L1LR | 0.864 (0.835-0.893) | 0.774 | 0.810 | 0.796 |
| LR_L1SVC | 0.860 (0.835-0.886) | 0.765 | 0.822 | 0.800 |
| LR_MIFS | 0.888 (0.863-0.912) | 0.792 | 0.834 | 0.819 |
| LR_PSCR | 0.899 (0.878-0.921) | 0.754 | 0.844 | 0.809 |
| LR_RECS | 0.848 (0.820-0.875) | 0.724 | 0.792 | 0.766 |
| LR_WLCX | 0.895 (0.871-0.919) | 0.760 | 0.828 | 0.804 |
| RF_FSCR | 0.812 (0.784-0.840) | 0.643 | 0.826 | 0.757 |
| RF_L1LR | 0.857 (0.834-0.880) | 0.676 | 0.844 | 0.780 |
| RF_L1SVC | 0.875 (0.852-0.898) | 0.686 | 0.858 | 0.793 |
| RF_MIFS | 0.802 (0.770-0.834) | 0.590 | 0.840 | 0.746 |
| RF_PSCR | 0.794 (0.765-0.823) | 0.572 | 0.836 | 0.736 |
| RF_RECS | 0.823 (0.792-0.854) | 0.646 | 0.846 | 0.770 |
| RF_WLCX | 0.802 (0.771-0.833) | 0.648 | 0.820 | 0.755 |

**Appendix 9 Figure captions**

**Figure S1. Portal venous phase computed tomography images of different scores based on the image quality assessment.** (a) - (b). Images with artifacts (nit nasointestinal tube and gas-fluid level, respectively; represent with red arrows). (c) - (f). Images with scores of 0 - 3 based on the degree of gastric filling.


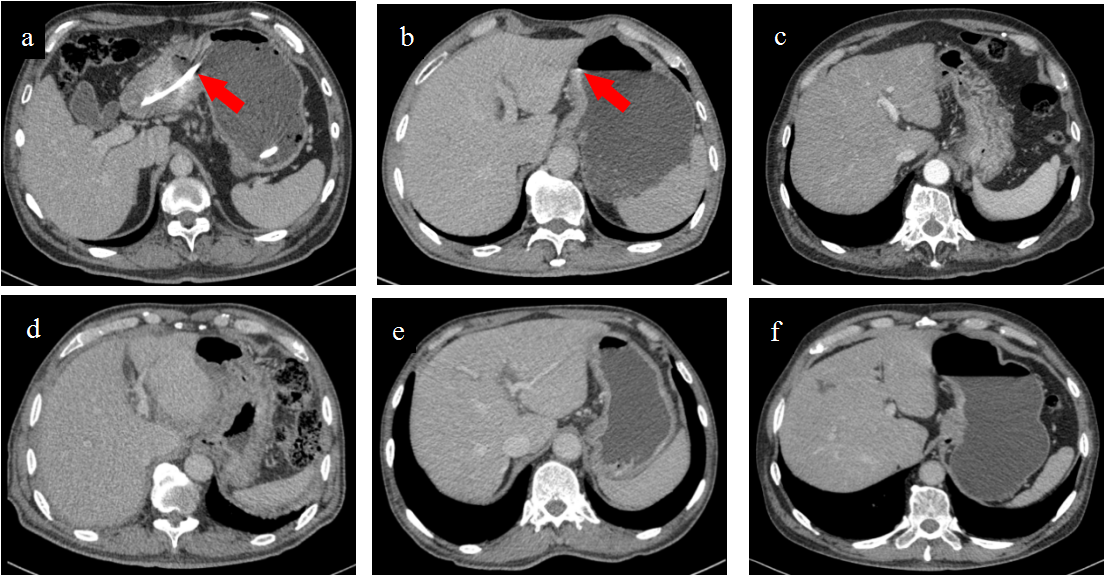


**Figure S2. The baseline and restaging CT images of a pDS patient.** Portal venous phase images of a female patient, at 47 years old with AGC at the stage of cT3N+M0, ypT1N1M0 after neo-adjuvant chemotherapy. The tumor area is outlined by a blue line.

NOTE. -CT = Computed tomography; pDS = pathological downstaging


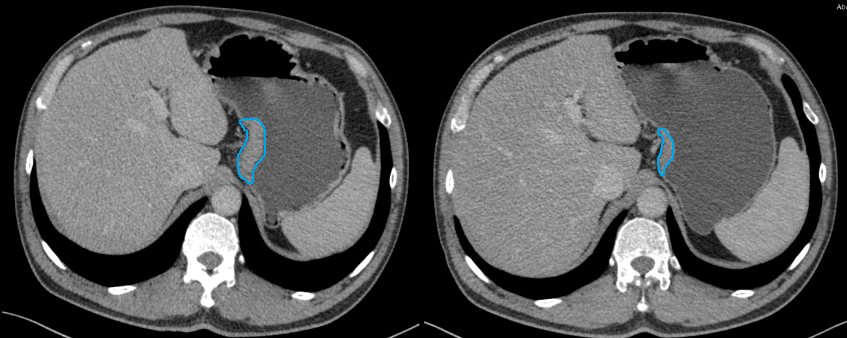


**Figure S3. The baseline and restaging CT images of a Non-pDS patient.** Portal venous phase CT images of a male patient, at 52 years old with AGC at the stage of cT4N+M0, ypT4N3M0 after neo-adjuvant chemotherapy. The tumor area is outlined by a blue line.

NOTE. -CT = Computed tomography; pDS = pathological downstaging


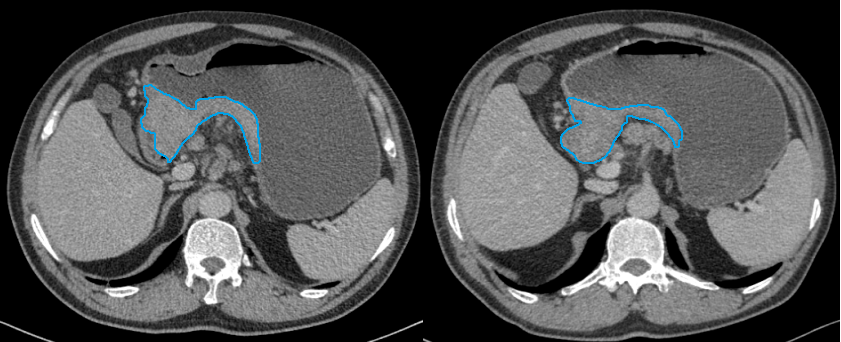


**Figure S4. The baseline and restaging CT images of a pDS patient with potential resactable tumor om admission.** Portal venous phase CT images of a male patient at 58 years old with AGC at the stage of cT4bN+M0, the fat gap between the tumor and the pancreas is not visible, and ypT0N0M0 after 2 cycles of neo-adjuvant chemotherapy. The red arrow shows fat gap.

NOTE. -CT = Computed tomography; pDS = pathological downstaging


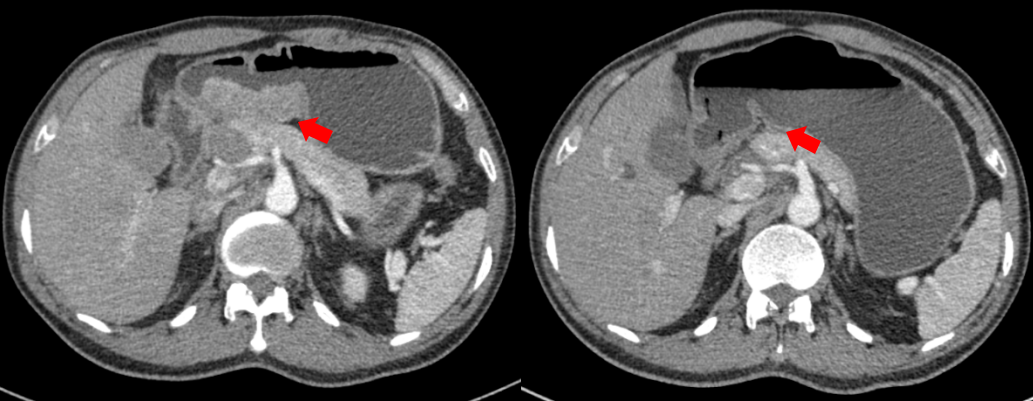


**Figure S5. The average AUC of the prediction and detective radiomics model varies with the number of features.** (a). The prediction radiomics model achieved the highest AUC value with 9 selected features in each cycle of the nested cross-validation structure. (b). The detection radiomics model achieved the highest AUC value with 79 selected features in each cycle of the nested cross-validation structure.

NOTE. -AUC = area under the curve


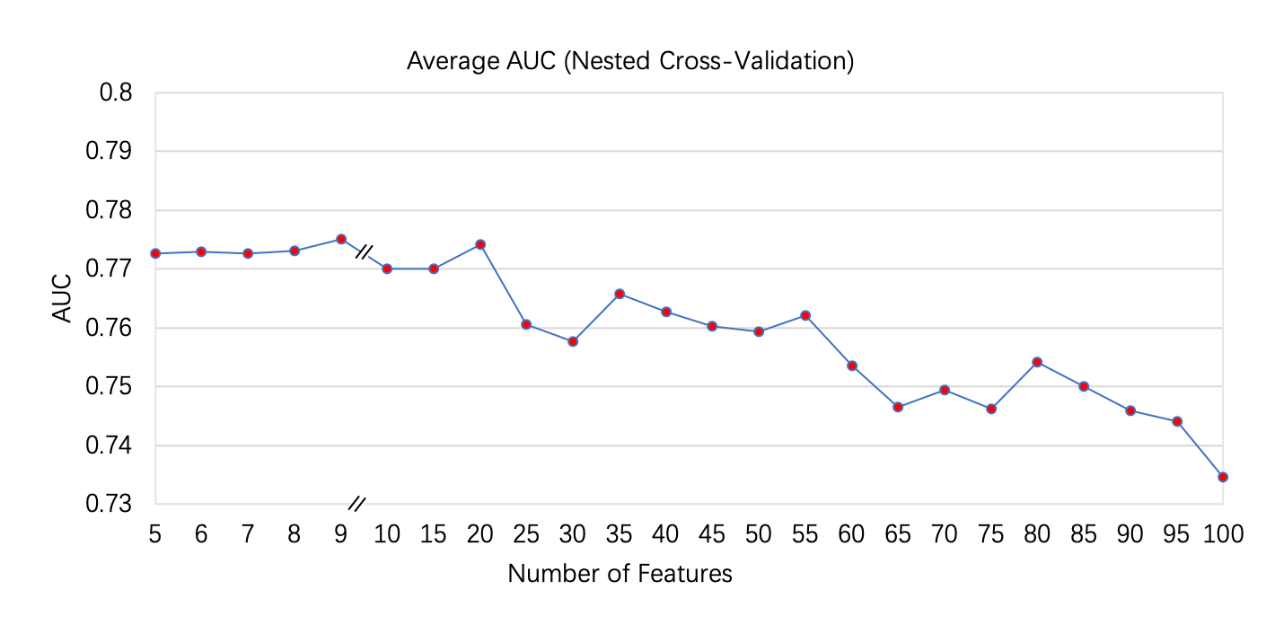


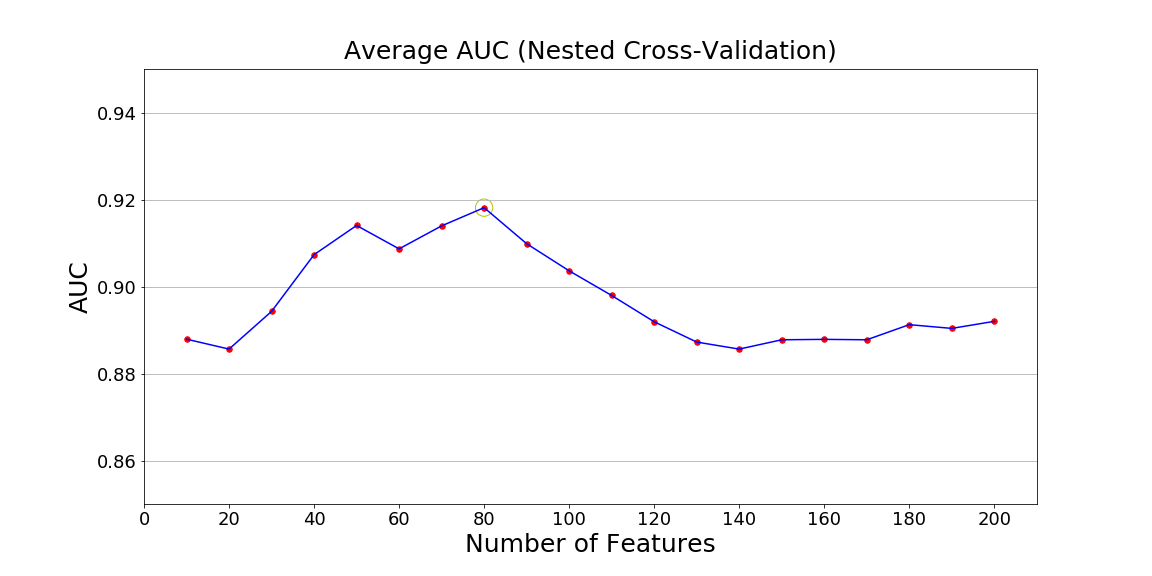


**Figure S6. The AUC, accuracy, sensitivity and specificity of the twenty-eight cross-combination machine-learning models.** Three of these models achieving high AUC ( > 0.900), of which were (a) the feature selection method of wilcoxon and classifier of linear SVC, (b) the feature selection method of F test and classifier of linear SVC and (c) the feature selection method of F-test and classifier of logistic regression. Among these three models, the optimal model consisted of the feature selector of wilcoxon and classifier of linearSVC.

NOTE. -AUC = area under the curve


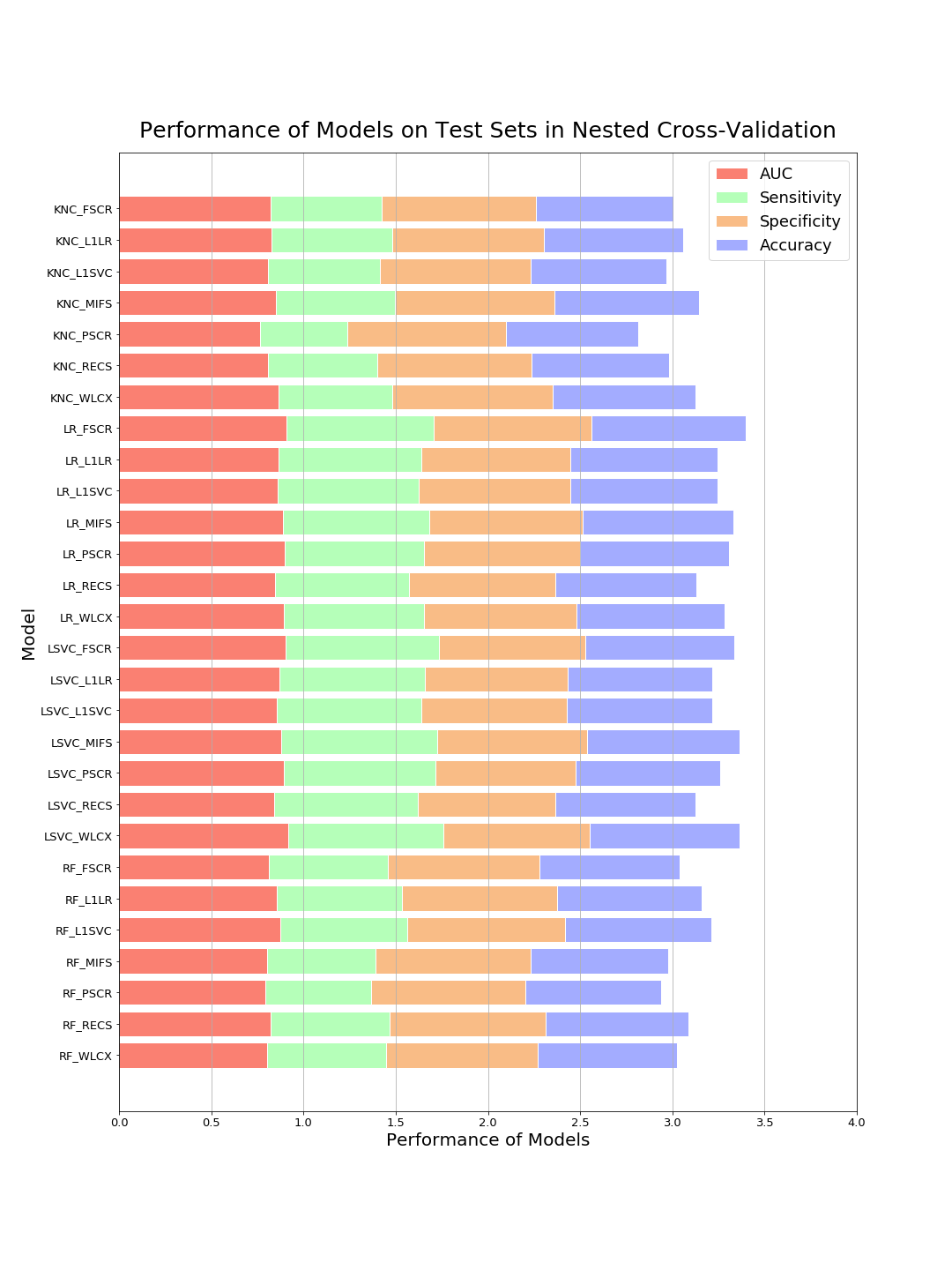

Supplement: Supplementary file 1 — (DOCX 3571 kb) [file 330_2021_7962_MOESM1_ESM.docx]
